# Subphenotyping of Mexican Patients With COVID-19 at Preadmission To Anticipate Severity Stratification: Age-Sex Unbiased Meta-Clustering Technique

**DOI:** 10.2196/30032

**Published:** 2022-03-30

**Authors:** Lexin Zhou, Nekane Romero-García, Juan Martínez-Miranda, J Alberto Conejero, Juan M García-Gómez, Carlos Sáez

**Affiliations:** 1 Biomedical Data Science Lab Instituto Universitario de Tecnologías de la Información y Comunicaciones Universitat Politècnica de València Valencia Spain; 2 Centro de Investigación Científica y de Educación Superior de Ensenada Ensenada Mexico; 3 Instituto Universitario de Matemática Pura y Aplicada Universitat Politècnica de València Valencia Spain

**Keywords:** COVID-19, subphenotypes, clustering, characterization, observational, epidemiology, Mexico

## Abstract

**Background:**

The COVID-19 pandemic has led to an unprecedented global health care challenge for both medical institutions and researchers. Recognizing different COVID-19 subphenotypes—the division of populations of patients into more meaningful subgroups driven by clinical features—and their severity characterization may assist clinicians during the clinical course, the vaccination process, research efforts, the surveillance system, and the allocation of limited resources.

**Objective:**

We aimed to discover age-sex unbiased COVID-19 patient subphenotypes based on easily available phenotypical data before admission, such as pre-existing comorbidities, lifestyle habits, and demographic features, to study the potential early severity stratification capabilities of the discovered subgroups through characterizing their severity patterns, including prognostic, intensive care unit (ICU), and morbimortality outcomes.

**Methods:**

We used the Mexican Government COVID-19 open data, including 778,692 SARS-CoV-2 population-based patient-level data as of September 2020. We applied a meta-clustering technique that consists of a 2-stage clustering approach combining dimensionality reduction (ie, principal components analysis and multiple correspondence analysis) and hierarchical clustering using the Ward minimum variance method with Euclidean squared distance.

**Results:**

In the independent age-sex clustering analyses, 56 clusters supported 11 clinically distinguishable meta-clusters (MCs). MCs 1-3 showed high recovery rates (90.27%-95.22%), including healthy patients of all ages, children with comorbidities and priority in receiving medical resources (ie, higher rates of hospitalization, intubation, and ICU admission) compared with other adult subgroups that have similar conditions, and young obese smokers. MCs 4-5 showed moderate recovery rates (81.30%-82.81%), including patients with hypertension or diabetes of all ages and obese patients with pneumonia, hypertension, and diabetes. MCs 6-11 showed low recovery rates (53.96%-66.94%), including immunosuppressed patients with high comorbidity rates, patients with chronic kidney disease with a poor survival length and probability of recovery, older smokers with chronic obstructive pulmonary disease, older adults with severe diabetes and hypertension, and the oldest obese smokers with chronic obstructive pulmonary disease and mild cardiovascular disease. Group outcomes conformed to the recent literature on dedicated age-sex groups. Mexican states and several types of clinical institutions showed relevant heterogeneity regarding severity, potentially linked to socioeconomic or health inequalities.

**Conclusions:**

The proposed 2-stage cluster analysis methodology produced a discriminative characterization of the sample and explainability over age and sex. These results can potentially help in understanding the clinical patient and their stratification for automated early triage before further tests and laboratory results are available and even in locations where additional tests are not available or to help decide resource allocation among vulnerable subgroups such as to prioritize vaccination or treatments.

## Introduction

In mid-January 2020, Mexico reported the first cases of COVID-19. In early March 2020, disease caused by SARS-CoV-2 was declared by the World Health Organization (WHO) as a pandemic [1]. As of August 13, 2020, a total of 20,439,814 confirmed cases of COVID-19 had been reported to the WHO, and 744,385 lives had been lost [2].

The COVID-19 pandemic has led to an unprecedented global health care challenge for both medical institutions and researchers. They have been making a huge effort to describe specific COVID-19 risk factor associations and severity outcomes, and personalized therapeutic options for COVID-19 patients are yet under investigation [3-5]. Recognizing different COVID-19 subphenotypes—the division of populations of patients into more meaningful subgroups driven by clinical features [6,7]—and their severity characterization may assist clinicians during the clinical course, research efforts, and the surveillance system. However, the availability of information to investigate such subphenotypes and consequent decision-making often varies both according to the stage at which patients are in the COVID-19 clinical workflow (eg, before admission, at admission, or during hospitalization) and according to hospital access possibilities (eg, hospitalized versus ambulatory patients), especially in locations where hospitalization is difficult. In addition, the patient age and sex entail a potential correlation between subgroup characterization and their severity characterization, which requires prudent usage in machine learning (ML) models.

Several studies have suggested potential COVID-19 subphenotypes, mainly within specific comorbidities such as pulmonary diseases or diabetes [8,9] or related to distinct genetic variants [10]. However, the Mexican population has its own particularity due to a high prevalence of comorbidities, like hypertension, diabetes—a leading cause of death in 2020 [11]—and obesity, which is leading the population to having undesirable risks for severe COVID-19 outcomes, higher than many other high-income countries [12]. Since distinct target populations often present with heterogeneous clinical characterization and severity outcomes, it remains crucial to gain a transparent understanding regarding the characterization of COVID-19 subphenotypes in Mexican patients to help anticipate individuals' prognostic outcomes if one gets infected and evaluate subphenotypic severity presentations.

Unsupervised ML is well-known for its usefulness in finding patterns in data [13,14]. We describe the results of an unsupervised ML meta-clustering approach to identify potential subphenotypes of COVID-19 patients in Mexico based on previously existing comorbidities, habits, and demographic features (ie, age and sex). Stratification on sex and age groups was included for 3 primary reasons: (1) to reduce potential ML models’ biases in best representing the majority (eg, young adults) but not underrepresenting other groups (eg, children and older adults) [15]; (2) to reduce potential confounding factors from age and sex, which are highly correlated with comorbidities, habits, and mortality (ie, age-sex clusters may help reveal more well-detailed patterns and phenotypical descriptions); and (3) to reduce interpretation biases (eg, if one healthy cluster presents a mortality rate of 98.5% but includes patients from all ages, this specific mortality rate may vary across 2 patients from the same cluster whose age differ significantly [eg, children versus adults]). See section 1 of Multimedia Appendix 1 for further details. In addition, we assessed the clusters’ source variability, namely the variability by Mexican states and types of clinical institutions (TCIs), to discern what types of clusters are prone to be in certain Mexican states or TCIs.

By using a population-based cohort of more than 700,000 patient-level cases, this is probably the largest cluster analysis about coronavirus patient-level cases to date. Other studies proposed unsupervised ML methods for aggregated population data [16], computed tomography image analyses [17,18], molecular-level clustering [19], or coronavirus-related scientific texts [20]. To date, several studies have provided results from unsupervised ML on patient-level epidemiological data [21-28]. To our knowledge, however, no characterized age-sex subphenotypes nor population-based studies with solely phenotypical information available at preadmission to aid automated risk stratification have been conducted, and neither characterized the Mexican population, which is generally more vulnerable due to its particularity of a high prevalence of comorbidities.

Performing accurate triage upon admission, especially in ambulatory settings, is often challenging, significantly depending on the patient information available to the physicians. This work, therefore, aimed to characterize age-sex unbiased COVID-19 subphenotypes that may potentially establish target groups for triage systems to assist clinicians in efficiently allocating limited resources and prioritize vaccination among subgroups who are more vulnerable when they get infected during the pandemic. As these subphenotypes are based on easily available data, such as previous disease and lifestyle habits, rather than COVID-19–related symptoms (eg, fever and nausea), vital signs, or biomarkers that are not often available in the first days of COVID-19 infection or difficult to obtain due to limited resources, our work therefore could support early triage prior to further tests and laboratory results and even provide guidance in areas where such tests are not available.

## Methods

### Data Collection and Processing

We used the data set collected by the General Epidemiology Directorate of the Mexican Ministry of Health, which is an open-source data set comprised of daily updated data from suspected COVID-19 cases (in public and private hospitals from all over the country), of which the positive cases were confirmed by laboratory tests for SARS-CoV-2 [29]. The data set is anonymized, open-access, and published by the Mexican government. The use of data followed the MX terms of free use of the Open Data of the Mexican Government [30]. As of November 2, 2020, the data set was comprised of a total of 2,414,882 cases, including patient-level demographic, comorbidity, habit, and prognosis data, for both positive and nonpositive cases. Noteworthy, the official website does not explicitly mention the source (each public and private health institution) for some of the information. Consulting with different health professionals, we concluded that it is more likely that every lab-confirmed patient with COVID-19 took a questionnaire in which the patients initially self-reported their comorbidities, and only those who were hospitalized were given a battery of tests to detect or confirm the highest-risk comorbidities, such as diabetes and hypertension.

We performed a series of data quality assessments such as detecting missing data and outliers, between-date inconsistency, erroneous data, and nonplausible data, and we also assessed potential temporal biases using temporal variability statistical methods [31]; no significant temporal changes were found (section 2 of Multimedia Appendix 1). However, we found that 95.28% of patients lost their lives within 31 days after the infection, which led us to remove the patients with symptom onset less than 31 days prior to the moment the data set was collected (ie, patients who showed symptoms after September 30, 2020) since these patients’ survival status in the future was still “unknown” (section 3 of Multimedia Appendix 1). Thus, patients infected after September 30, 2020, were excluded.

**Figure 1:**
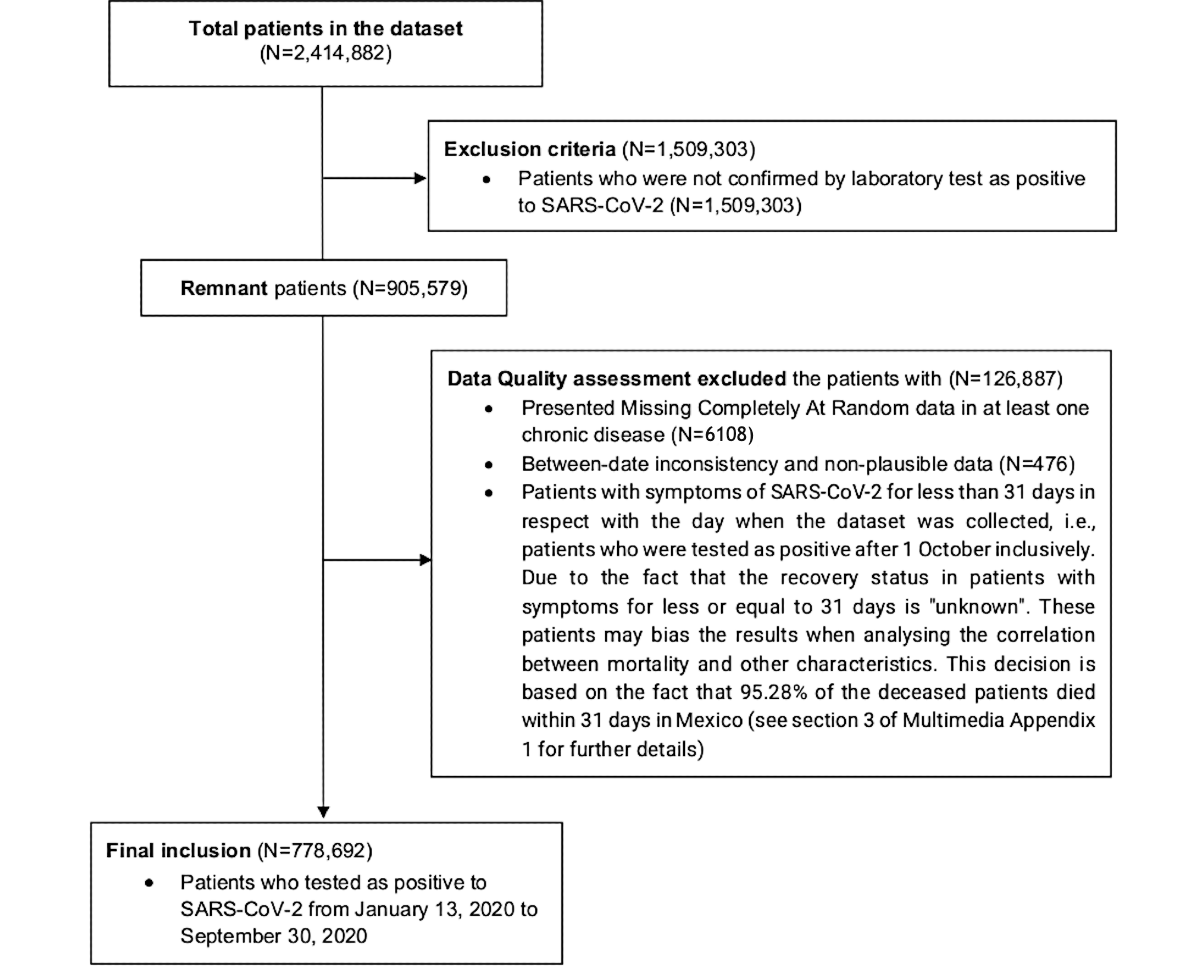
Data set preprocessing flowchart for data in Mexico from January 13, 2020 to September 30, 2020.

Figure 1 describes the study inclusion and exclusion criteria and the data quality assessment process outcomes in a CONSORT-like flowchart. The final sample included 778,692 positive cases.

### Study Measures

We derived 6 outcome variables related with the prospective patient’s severity. The first was the patient outcome (deceased or not) from the date of death record. The second was the number of days from symptom onset to hospital admission. Third, we categorized 2 variables describing the overall survival at 15 days and 30 days after symptom onset. Lastly, we categorized 2 variables that also described the overall survival at 15 days and 30 days after symptom onset but only for the deceased patients.

Table 1 shows the list of studied variables. Sections 4 to 6 in Multimedia Appendix 1 describe additional information on the original data set, baseline characteristics of the COVID-19 patients alongside descriptive statistics in age-sex groups of the study sample, and association between pregnancy and outcomes.

**Table 1:** List of variables contained in the data set for the study cases; they were originally coded in Spanish and translated into English by the authors for this work. Section 4 in Multimedia Appendix 1 provides the description of the original data set.

Variable	Description	Type (value/format)
Sex	Sex of the person (defined in the metadata published by the Mexican government)	Discrete (Male, Female)
Age	Age in years at the time of the admission	Numerical integer
Pregnant	Presence of pregnancy	Discrete (Yes, No)
Obesity	Presence of obesity	Discrete (Yes, No)
Smoke	Presence of smoking habit	Discrete (Yes, No)
Pneumonia	Presence of pneumonia	Discrete (Yes, No)
Diabetes	Presence of diabetes	Discrete (Yes, No)
COPD^a^	Presence of chronic obstructive pulmonary disease	Discrete (Yes, No)
Asthma	Presence of asthma	Discrete (Yes, No)
INMUSUPR^b^	Presence of immunosuppression	Discrete (Yes, No)
Hypertension	Presence of hypertension	Discrete (Yes, No)
CKD^c^	Presence of chronic kidney disease	Discrete (Yes, No)
Cardiovascular	Presence of cardiovascular	Discrete (Yes, No)
Other disease	Presence of other diseases	Discrete (Yes, No)
Hospitalized	Whether a patient was hospitalized or ambulant	Discrete (Yes, No)
Intubated	Whether a patient was intubated	Discrete (Yes, No)
ICU^d^	Whether a patient had been in an intensive care unit	Discrete (Yes, No)
Other case contact	Whether a patient was detected to have contact with other coronavirus cases	Discrete (Yes, No)
Result_lab	Coronavirus test result	Discrete (Positive SARS-CoV-2, Non-Positive SARS-CoV-2, Pending, Inadequate result, Not Applied)
Admission_date	The date when a patient attended the care unit (not necessarily hospitalized)	Date (dd/mm/yyyy)
Symptoms_date	The date of symptom onset	Date (dd/mm/yyyy)
Death_date	The date of death	Date (dd/mm/yyyy)
Entity_um	The state where a patient received attention from a medical unit	Discrete
TCI^e^	The type of institution in the National Health System that provided medical care	Discrete^f^
Outcome^g^	Death result of the patient (we used this to calculate mortality and recovery rate)	Discrete (Deceased, Non-Deceased)
Survival>15days^g^	Whether a patient survived more than 15 days from symptoms onset	Discrete (Yes, No)
Survival>30days^g^	Whether a patient survived more than 30 days from symptoms onset	Discrete (Yes, No)
Survival>15days_deceased^g^	Whether a deceased patient survived more than 15 days from symptom onset	Discrete (Yes, No)
Survival>30days_deceased^g^	Whether a deceased patient survived more than 30 days from symptom onset	Discrete (Yes, No)
From Symptom to Hospital days^g^	The days that it took between symptom onset and hospitalization	Numerical integer

^a^COPD: chronic obstructive pulmonary disease.

^b^INMUSUPR, immunosuppression.

^c^CKD: chronic kidney disease.

^d^ICU, intensive care unit.

^e^TCI: type of clinical institution.

^f^IMSS (Mexican Institute of Social Security), SSA (Secretariat of Health), ISSSTE (Institute for Social Security and Services for State Workers), PRIVATE, PEMEX (Mexican Petroleum Institution), STATE, SEMAR (Secretariat of the Navy), SEDENA (Secretariat of the National Defense), IMSS-BIENESTAR, UNIVERSITARY, MUNICIPAL, RED CROSS, DIF (National System for Integral Family Development).

^g^Variables that were created by combining or transforming other variables in the original data set.

### Meta-Clustering Methodology

**Figure 2:**
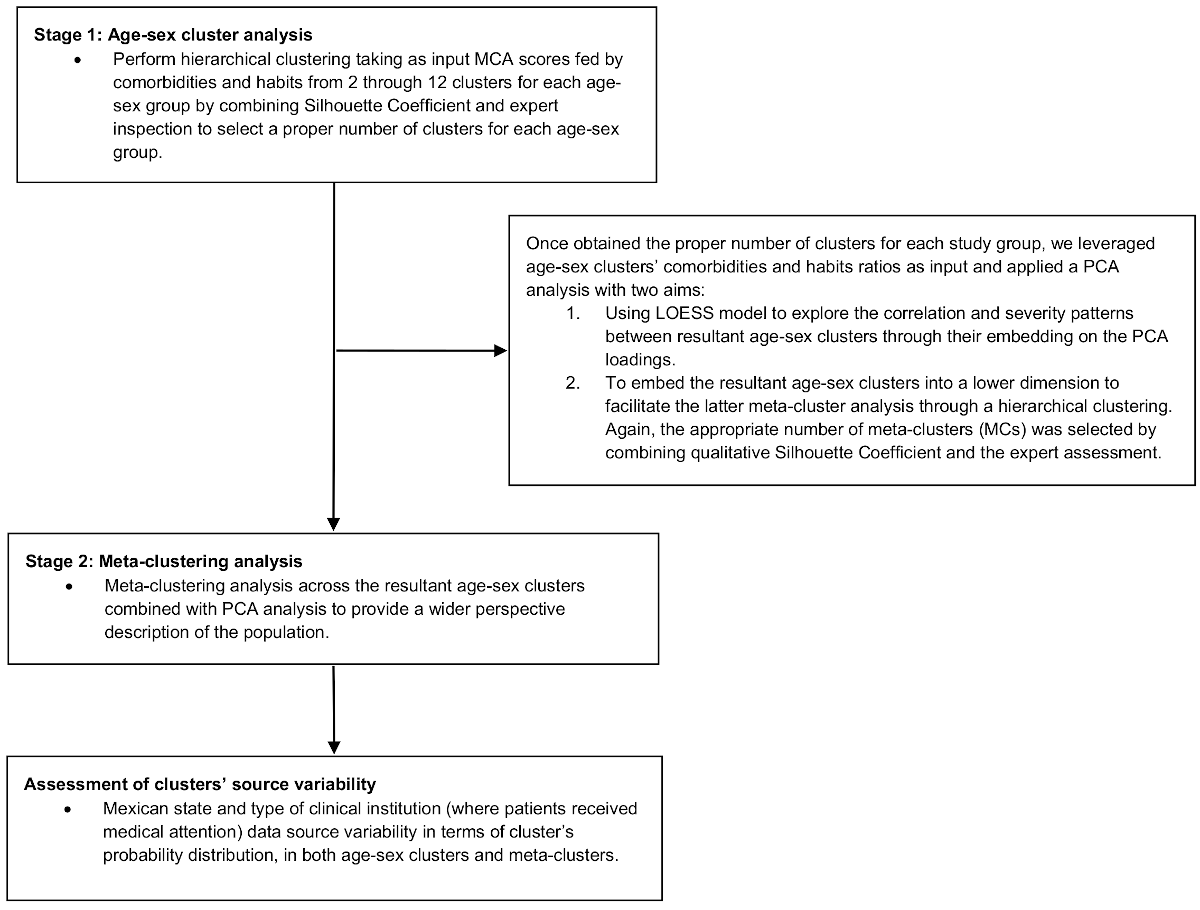
Research methodology flowchart. LOESS: locally estimated scatterplot smoothing; MCA: multiple correspondence analysis; PCA: principal component analysis.

We applied a 2-stage subgroup discovery approach (Figure 2 summarizes the full methodology). In both stages, we used the Ward minimum variance method with Euclidean squared distance [32] to perform hierarchical clustering fed by a dimensionality reduction algorithm—principal component analysis (PCA) or multiple correspondence analysis (MCA) [33,34]—that took as input 11 variables including 9 comorbidities—pneumonia, diabetes, chronic obstructive pulmonary disease (COPD), asthma, immunosuppression, hypertension, chronic kidney disease (CKD), cardiovascular disease, and other diseases—alongside 2 unhealthy habits, namely obesity and smoking. In order to select the most representative PCA and MCA components to feed the hierarchical clustering, we considered values with an eigenvalue higher than the average. Dimensionality reduction is known to help in the process of clustering by compressing information into a smaller number of variables, making unsupervised learning less prone to overfitting [35], as well as to facilitate further visual analytics to prevent the potential ML black-box issue [36].

In the first stage, we used the entire data set—778,692 patients—since unsupervised ML does not require splitting the data into training and test data [13,14]. We applied individually hierarchical clustering analyses, taking as the input the MCA scores fed by comorbidities and habits for the stratified groups according to sex and age (<18, 18-49, 50-64, and >64 years) to reduce potential biases and confounding factors, since age and sex are highly correlated with comorbidities, habits, and mortality. Then, we applied PCA and a locally estimated scatterplot smoothing (LOESS) model [37] to the resultant age-sex clusters’ features to visually explain their correlations and severity relationships. We created a cluster heat map to help understand the characteristics of each age-sex cluster.

In the second stage, in a population description providing a wider perspective, we performed a hierarchical clustering again fed by PCA scores obtained via the resultant age-sex clusters, taking as input their comorbidities and habits ratios. Then, we quantified the features of the resultant meta-clusters (MCs) via a table and summarized these quantified features into a qualitative table to help interpret the main features of the resultant MCs.

For each subgroup analysis, we implemented cluster analyses from 2 through 12 clusters. The proper number of subgroups was obtained by combining a quantitative approach using the silhouette coefficient [38]—which measures the tightness and separation of the objects within clusters, reflecting how similar an object is to its own cluster compared with other clusters—and a qualitative cluster analysis audited by the authors of this work, including medical, health informatics, and ML experts from Spain and Mexico. We first selected the group of clusters that showed relatively better silhouette coefficient values, then adjusted the number for the most reasonable and clinically distinguishable groups regarding clinical phenotypes. This process was supported by the pipelines and exploratory tool we developed in previous work [39,40].

Finally, we performed a source variability assessment [41] using heat maps to analyze the severity tendency among different data sources based on the clusters’ probability distributions between Mexican states and several TCIs where patients received medical attention.

Data processing and analyses were performed using RStudio (version 3.6) and Python (version 3.8). Temporal and source variability—data quality analyses—were performed using the EHRtemporalVariability [31] and EHRsourceVariability [41-43] packages. Further information about the methods and codes that support the findings of this study are available in section 7 of Multimedia Appendix 1.

## Results

### Age-Sex Cluster Analysis

After evaluating the stratified clustering results, we selected the following number of clusters (k) for each specific age-sex group: <18-Male: k=5; <18-Female: k=4; 18-49-Male: k=7; 18-49-Female: k=7; 50-64-Male: k=9; 50-64-Female: k=8; >64-Male: k=8; >64-Female: k=8. This resulted in 56 age-sex clusters in total. Section 8 of Multimedia Appendix 1 provides the number of individuals for each age-sex cluster. The second-stage meta-clustering analysis uncovered 11 clinically distinguishable MCs among the 56 age-sex clusters.

**Figure 3:**
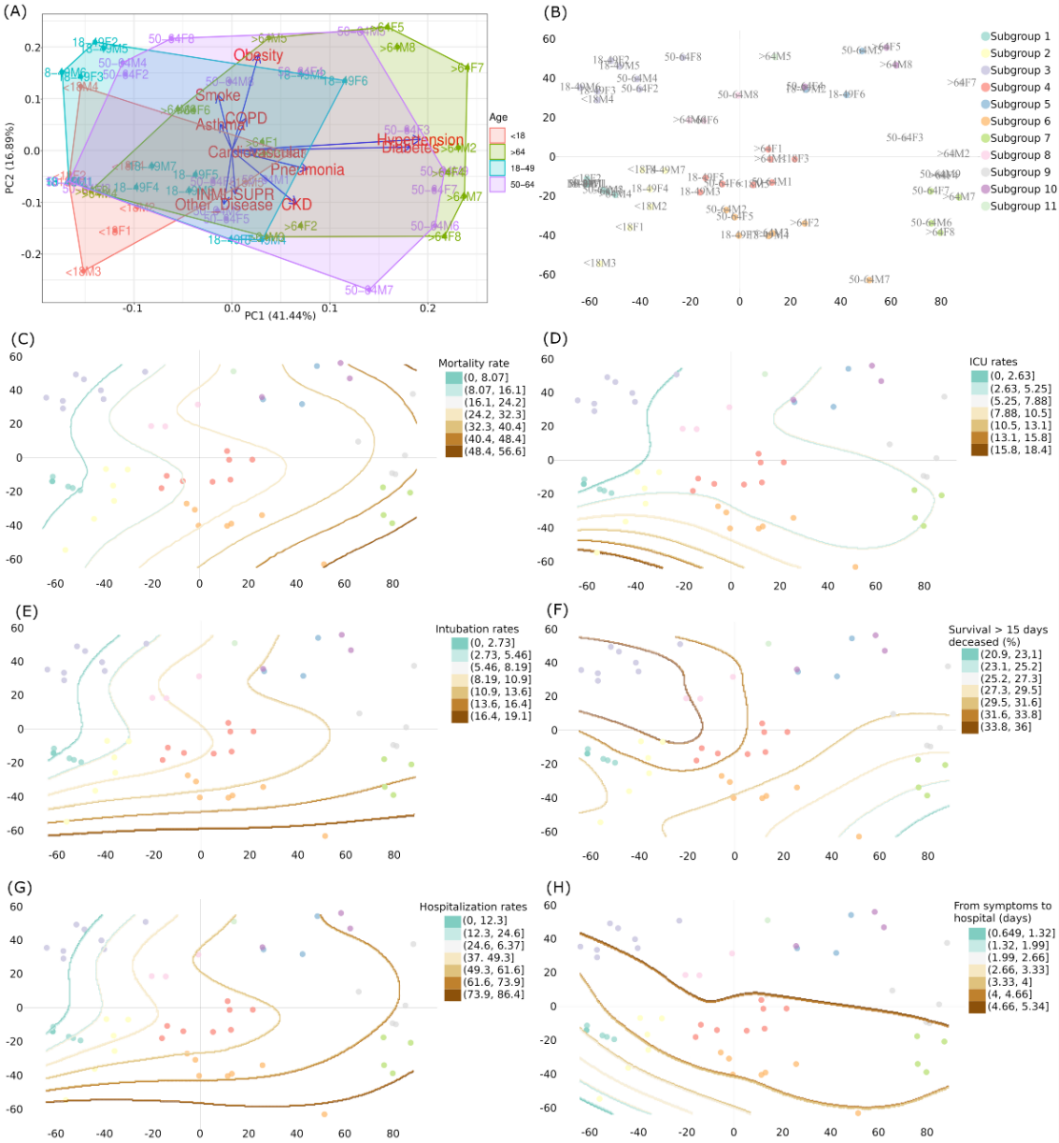
Principal component analysis (PCA) of the 56 age-sex clusters, meta-clustering results, and locally estimated scatterplot smoothing (LOESS)–based delineations for 7 severity ranges: (A) PCA from 56 age-sex stratified clusters; (B) scatterplot of the 11 meta-clusters (MCs) defined from the 56 clusters; (C) LOESS scatterplot for mortality; (D) LOESS scatterplot for intensive care unit (ICU) admission; (E) LOESS scatterplot for intubation; (F) LOESS scatterplot for survival at 15 days among deceased patients; (G) LOESS scatterplot for hospitalization; and (H) LOESS scatterplot for days from symptom onset to hospitalization. All the scatter plots share coordinates. Each subgroup is denoted using the following abbreviation: [AgeGroup][Sex][ClusterID].

Figure 3 describes the relationships among different comorbidities and habits in the original 56 age-sex clusters through the first 2 principal components (Figure 3A). It also provides the correspondence to their assigned MCs (Figure 3B) and their LOESS delineations for distinct severity outcomes (Figure 3C, Figure 3D, Figure 3E, Figure 3F, Figure 3G, and Figure 3H).

The PCA uncovered noticeable patterns and characterizations among the clusters representing different ages in both sexes. Young adults are prone to asthma and habitual smoking, whereas older adults are prone to many comorbidities such as hypertension, diabetes, obesity, COPD, pneumonia, and CKD. The results also show that obesity and habitual smoking—both positively correlated—are strongly separated from immunosuppression and other diseases, which are both positively correlated.

The LOESS models showed that children had fewer days between symptom onset and hospitalization and higher rates of intensive care unit (ICU) admission, intubation, and hospitalization than adults with similar conditions (Figure 3D, Figure 3E, Figure 3G, and Figure 3H). In contrast, MC3 (a young obese cluster with moderate asthma and smoking rates) behaved inversely.

Inspecting the relationship between the PCA and LOESS models showed that CKD was significantly associated with a shorter survival length among deceased patients and an increase in intubation rates (Figure 3E, Figure 3D). Mortality constantly increased from children to older adults, but the most severe zones were those for pneumonia, CKD, and COPD (Figure 3C), independent of the age groups.

**Figure 4:**
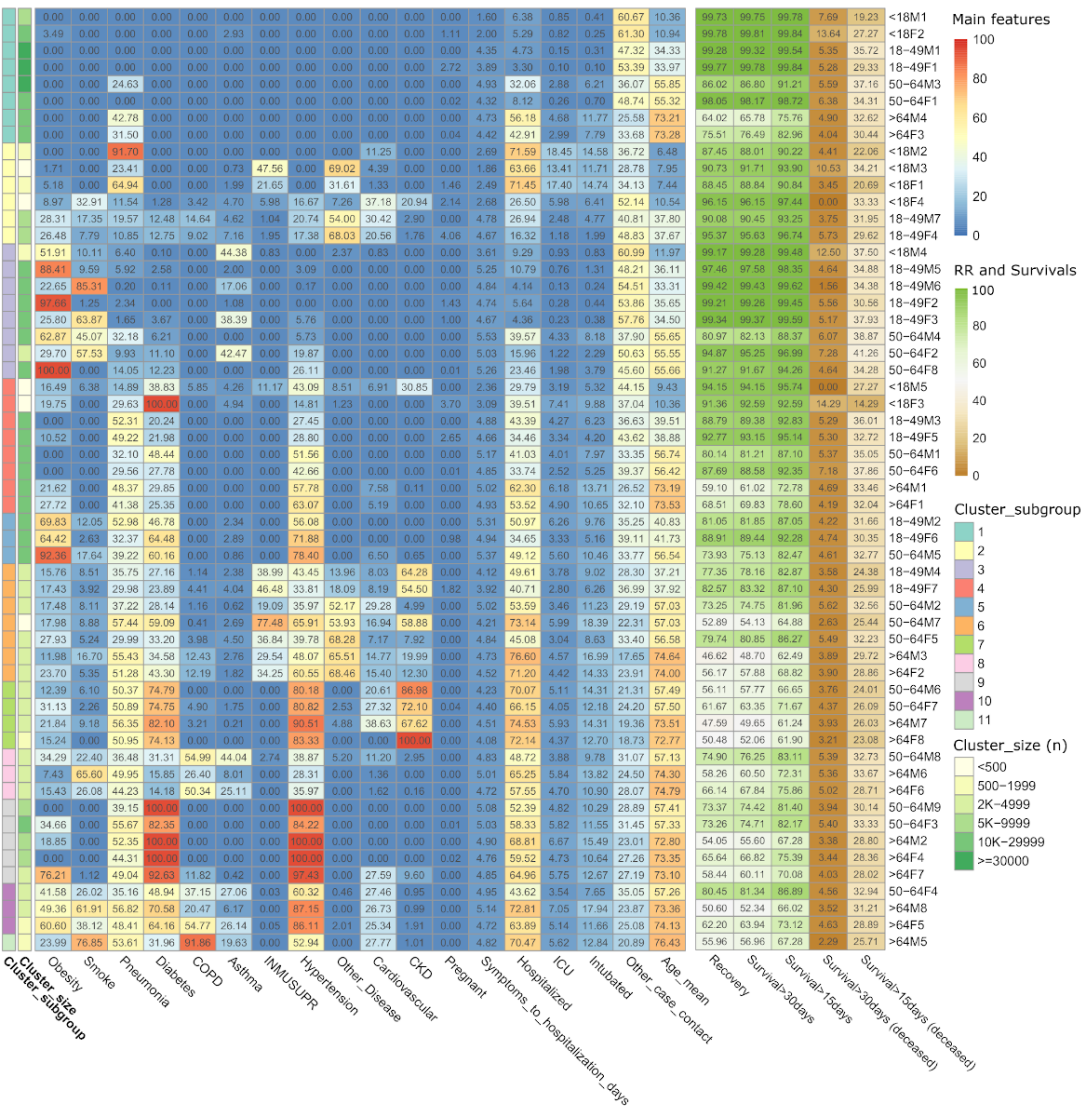
Heat map showing the quantified characteristics among 56 age-sex–specific clusters of the 11 meta-clusters (MCs) of data collected in Mexico between January 13, 2020 and September 30, 2020.; the size of each cluster (n) was categorized into 6 ranges. CKD: chronic kidney disease; COPD: chronic obstructive pulmonary disease; ICU: intensive care unit; RR: recovery rate.

Figure 4 describes and quantifies the features of the 56 age-sex clusters and relates them to their MCs. Figure 4 reinforces that children had a faster time from symptom onset to hospitalization and were prone to ICU admission despite presenting with a similar clinical condition as adults (eg, cluster <18M3 versus 50-64F5). Regarding sex discrepancies, female patients showed a better recovery rate (RR) despite presenting with similar clinical conditions as male patients (eg, >64M1 versus >64F1).

### Meta-Clustering Analysis

**Figure 5:**
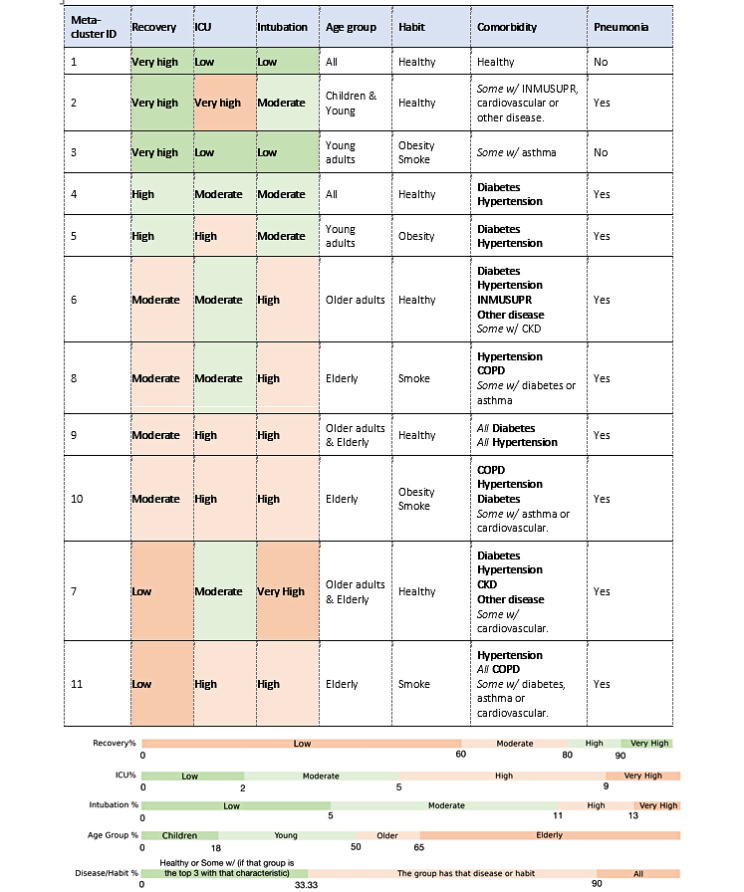
Main features of the 11 resultant meta-clusters, sorted by recovery, in addition to the thresholds for the different severity outcomes and input variable categories; based on data collected in Mexico between January 13, 2020 and September 30, 2020. COPD: chronic obstructive pulmonary disease; ICU: intensive care unit; INMUSUPR: immunosuppression.

Table 2 represents the quantified features of the 11 resultant MCs. Figure 5 summarizes the main features of the 11 resultant MCs. Variable values were categorized according to clinical meaningful thresholds proposed by the authors as defined in the table legend. Next, we describe the clinically distinguishable main epidemiological findings for each MC.

MC1 included the 2 healthiest clusters per each age-sex group, with a very high RR (90%). Most deceased patients in MC1 with pneumonia were older patients (Figure 4). MC2 included children and young individuals (mean age 18 years) with healthy habits and little incidence of relevant diseases (13% immunosuppression, 17% cardiovascular disease, 4% CKD), albeit the RR was very high (91%). In addition, MC2 had the highest ICU admission rate (9%), driven by 3 child clusters whose ICU rates varied from 13.41% to 18.45%. MC3 included young adults (mean age 40 years) with significant obesity and smoking as well as a low incidence of other diseases and very high RR (95%). Despite the similarly high RRs in MC1 to MC3, MC1 and MC3 showed a low incidence of pneumonia, while one-third of the patients in MC2 had pneumonia.

**Table 2:** Distribution of age, features, and comorbidities with the quantitative description of demographic features, treatment, and epidemiological characteristics among the 11 meta-clusters (MCs) based on the arithmetic mean presuming that each age-sex cluster is representative of its population; thus, the size (n) of each age-sex cluster was ignored.

Characteristics		MC1	MC2	MC3	MC4	MC5	MC6	MC7	MC8	MC9	MC10	MC11
Age-sex clusters (total n=56), n		8	6	8	8	3	7	4	3	5	3	1
Patients in the MC (total n=778,892), n		407,005	13,826	11,3537	11,1950	42280	21642	9239	9687	40557	7777	1192
**Demographics**												
	Age (years), mean	43.4	18	39.8	44.8	46.4	56.3	65.3	68.7	66.8	68.2	76.4
	Female sex, %	50	50	50	50	33.33	42.86	50	33.33	60	66.67	0
**Age range (years), %**												
	<18	25	66.67	12.5	25	0	0	0	0	0	0	0
	18-49	25	33.33	50	25	66.67	28.57	0	0	0	0	0
	50-64	25	0	37.5	25	33.33	42.86	50	33.33	40	33.33	0
	>64	25	0	0	25	0	28.57	50	66.67	60	66.67	100
Pregnancy (yes), %		0.49	1.28	0.3	0.8	0.33	0.26	0.01	0	0.01	0	0
**Habits, %**												
	Obesity	0.44	11.78	59.88	12.01	75.54	18.89	20.15	19.05	25.94	50.51	23.99
	Smoker	0	9.67	34.09	0.8	10.77	8.1	4.38	38.03	0.22	42.02	76.85
**Comorbidities, %**												
	Diabetes	0	4.42	4.5	39.06	57.14	35.62	76.44	20.45	95	61.23	31.96
	COPD^a^	0	4.51	0	0.73	0	5.1	2.03	43.91	2.36	37.46	91.86
	Asthma	0.37	3.2	18.17	1.15	2.03	2.69	0.49	25.72	0.08	19.79	19.63
	INMUSUPR^b^	0	13.03	0.1	1.4	0	40.38	0	0.91	0	0.03	0
	Hypertension	0	9.13	7.59	41.15	68.79	46.79	83.71	34.38	96.33	77.86	52.94
	Other disease	0	38.32	0.3	1.22	0	48.63	1.85	1.73	0	0.82	0
	Cardiovascular	0	17.52	0.1	2.46	2.17	14.25	21.64	4.73	5.52	26.51	27.77
	CKD^c^	0	4.27	0	3.87	0.22	31.84	81.67	1.04	1.92	1.28	1.01
**Treatment, %**												
	Hospitalized	19.87	46.08	14.15	42.22	44.91	58.56	70.72	57.17	60.8	60.11	70.47
	ICU^d^	1.59	9.82	1.23	4.48	5.06	4.01	4.87	4.81	5.56	5.24	5.62
	Intubated	3.44	9.03	2.18	7.9	8.46	12.12	13.38	11.5	12.13	12.42	12.84
Pneumonia, %		12.36	37	9.08	37.18	41.52	42.44	52.14	43.55	48.1	46.8	53.61
Recovery, %		90.27	91.37	95.22	82.81	81.3	66.94	53.96	66.43	64.95	64.42	55.96
Survival >15 days, %		93.46	93.73	97.01	88.39	87.27	76.34	65.37	77.1	75.26	75.34	67.28
Survival >30 days, %		90.74	91.8	95.5	83.74	82.14	68.26	55.71	68.2	66.33	65.88	56.96
Survival >15 days (deceased), %		30.76	28.64	36.21	31.09	31.59	28.46	24.8	31.7	29.73	31.01	25.71
Survival >30 days (deceased), %		6.61	4.64	5.93	5.79	4.52	4.2	3.82	5.26	4.04	4.24	2.29
Time from symptom onset to hospitalization (days), mean		3.78	3.2	4.87	4.37	5.21	4.48	4.3	4.85	4.92	4.94	4.82
Other case contact, %		45.84	40.23	51.18	36.6	36.04	27.39	20.9	27.88	27.56	28	20.89

^a^COPD: chronic obstructive pulmonary disease.

^b^INMUSUPR: immunosuppression.

^c^CKD: chronic kidney disease.

^d^ICU: intensive care unit.

MC4 included individuals of all ages with healthy habits, but, unlike MC1, most patients in MC4 had hypertension (41%) or diabetes (39%), but not both simultaneously. MC5 included young adults with obesity (75%), diabetes (57%), or hypertension (69%). Despite this dissimilarity, MC4 and MC5 still had similarly high RRs, of approximately 80%. From MC4 onwards, all MCs had a 40% to 50% incidence of pneumonia, as provided on the case report, which does not exclude the possibility that some patients developed pneumonia days after. Noteworthy, in MC4 to MC11, more than 70% of the deceased patients were diagnosed with pneumonia.

The RRs from MC6 and MC8-MC10 were similar (64%-67%). MC6 included older adults with no obesity nor smoking but with a high prevalence of diseases including diabetes, hypertension, immunosuppression, or others. MC8 included older adults who habitually smoked, plus hypertension (34%) or COPD (44%), both smoking-related diseases. Similarly, MC10 included older adults with obesity (50%) or habitual smoking (42%), who also had COPD (37%) but with a much higher incidence of diabetes (61%) and hypertension (78%). MC9 included older adults with both diabetes (95%) and hypertension (96%).

MC7 and MC11 had the lowest RRs (54% and 56%, respectively). MC7 included older adults with common diseases (diabetes, hypertension, and cardiovascular disease) plus CKD (81%). CKD stands out as the differential factor between similar MCs with low RRs, such as MC6 or MC9. MC11 was similar to MC8 and MC10; the key differences were the higher prevalence of smoking (78%, which doubles the former) and COPD (almost all patients, 91%) and a mean age 8 years older (76 years versus 68 years). In addition, MCs that included older obese patients who habitually smoked (MC8, MC10, and MC11) had significantly higher COPD and cardiovascular disease incidences, associations that did not occur with the young smokers (MC3).

### Assessment of Clusters’ Source Variability

**Figure 6:**
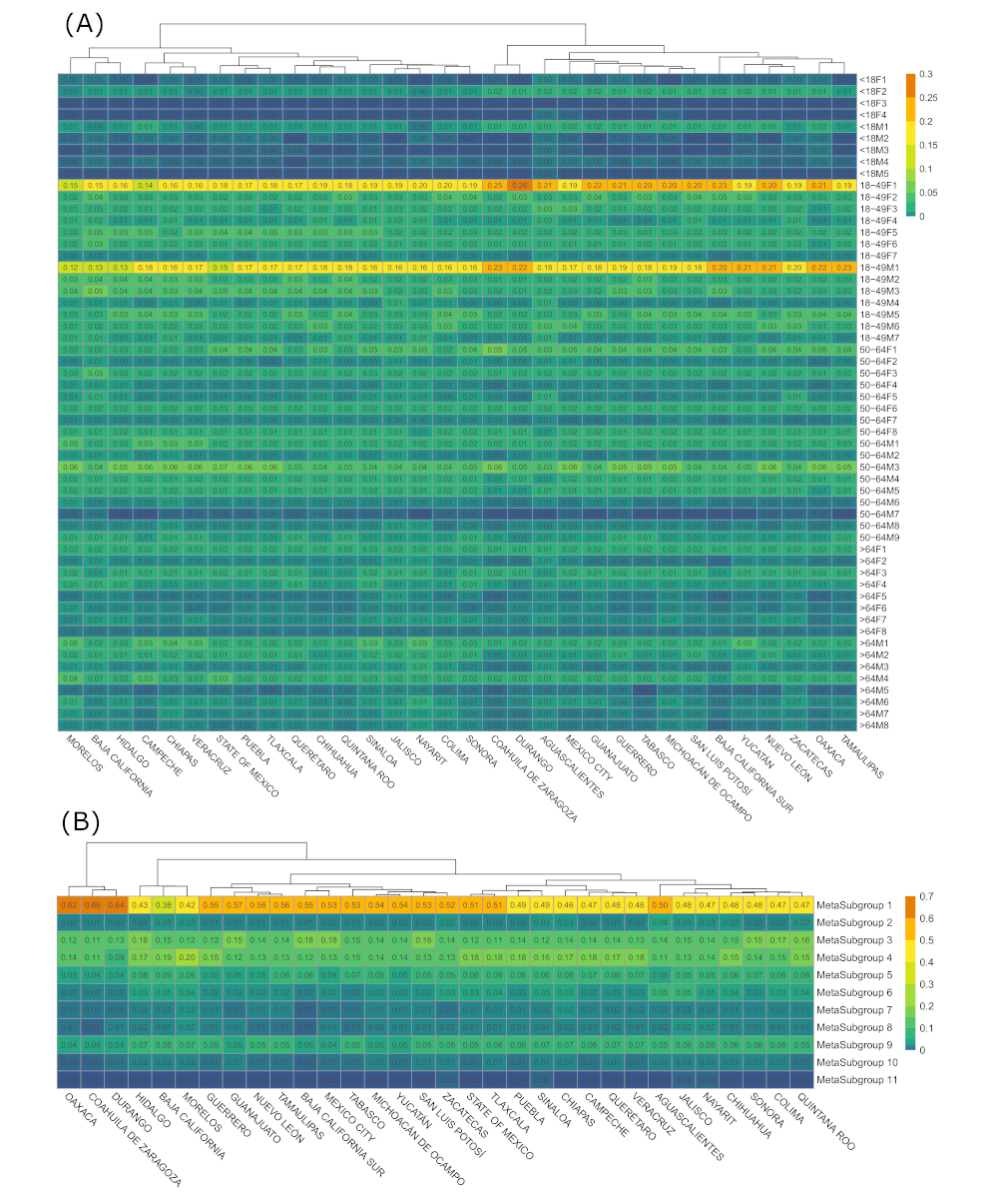
Heat maps of the probability distribution of the (A) 56 age-sex specific clusters and (B) 11 meta-clusters (MCs) for each Mexican state where patients received treatment or medical attention, using data collected in Mexico between January 13, 2020 and September 30, 2020. Rows represent the clusters, and columns represent the states and are arranged according to a hierarchical clustering of their values. We compared the clusters' distribution within each age range to circumvent any correlation or association with comorbidities and habits.

Regarding state variability, half of the Mexican states were prone to higher probabilities of healthy clusters with better RRs, lower hospitalization rates, lower ICU rates, and lower intubation rates among each age-sex group (Figure 6A; eg, 18F2, 18M1, 18-49F1, 18-49M1, 50-64F1) and MCs (Figure 6B), whereas another one-half behaved inversely. Hidalgo, Baja California, and Morelos represented the healthiest groups, in contrast to Oaxaca, Coahuila de Zaragoza, and Durango, which represented the less healthy. Surprisingly, Mexico City showed a significantly higher probability of having healthier clusters than the State of Mexico, albeit the populations of their main urban areas are close, and both have similar resources and economic development levels.

**Figure 7:**
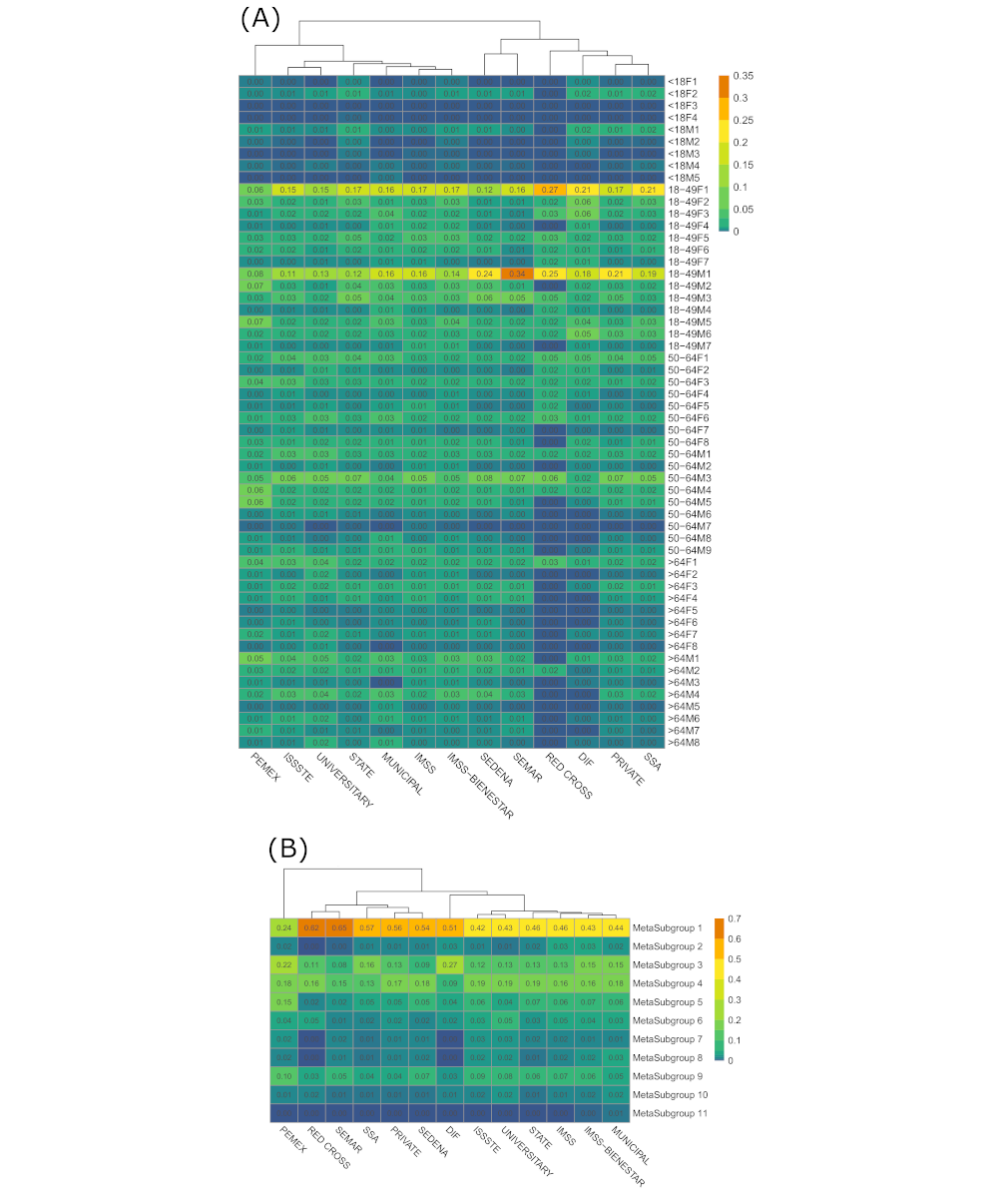
Heat maps of the probability distribution of the (A) 56 age-sex specific clusters and (B) 11 MCs for each type of clinical institution (TCI), using data collected in Mexico between January 13, 2020 and September 30, 2020. Rows represent the clusters, and columns represent the TCIs and are arranged according to a hierarchical clustering of their values. We compared the clusters' distribution within each age range to circumvent any correlation or association with comorbidities and habits. DIF: National System for Integral Family Development; IMSS: Mexican Institute of Social Security; ISSSTE: Institute for Social Security and Services for State Workers; PEMEX: Mexican Petroleum Institution; SEDENA: Secretariat of the National Defense; SEMAR: Secretariat of the Navy; SSA: Secretariat of Health.

Regarding variability in the TCIs (Figure 7A, Figure 7B), the Secretariat of Health (SSA), the National System for Integral Family Development (DIF), private institutions, and the Red Cross were more likely to have healthier, young patients. This pattern occurred inversely in other TCIs, especially the Mexican Petroleum Institution, for which the probabilities of severe clusters were generally higher. The clinical institutions of the armed forces (Secretariat of the Navy [SEMAR], Secretariat of the National Defense [SEDENA]) were mostly healthy, intuitively with a higher probability of male patients. Noteworthy, among the 3 primary TCIs in Mexico, the public health system (SSA) had more mild comorbidities and relatively higher probabilities of having healthy clusters among each age-sex group, mostly in MC1 (57%) and MC3 (16%), whereas in the 2 main social security systems (Mexican Institute of Social Security [IMSS], Institute for Social Security and Services for State Workers [ISSSTE]), the situation was just the opposite.

## Discussion

### Meta-Clustering

#### Main Findings

Previous literature has reported isolated risk factors and their association with severe progression of several diseases. However, the use of such information to improve clinical decision-making is potentially limited. In this work, no single clinical variable nor lifestyle habit was enough to characterize the COVID-19 subphenotypes, a typical phenomenon when the data set has many categorical variables. This reflects the reality of clinical practice: Patients do not usually fall into subgroups of “all good” or “all bad” outcomes, and neither of the patient outcomes can be concluded by a single variable. However, when considering the variables together, our study uncovered 11 clinically distinguishable MCs among 56 plausible age-sex clusters; these MC-defined subphenotypes alongside age-sex stratification may represent different disease mechanisms and outcomes.

Each of the 11 MCs shows clinical consistency: Their group outcomes can be potentially predicted from the proposed input variables, according to the literature published to date. From an outcome perspective, a dividing line can be clearly drawn between MCs 1-5 and MCs 6-11. Although the former had high RRs, the latter had low RRs. Several factors can explain these findings, mainly age, habits, and comorbidities. Since all MCs were 30%-60% women within their input age-sex clusters, the association between sex and mortality is hard to see based only on MCs. However, the age-sex cluster analysis showed clearly better outcomes in female patients despite presenting with similar conditions as male patients. Therefore, considering both age-sex clusters and MCs is essential for better characterization that reveals more relevant detailed information in COVID-19 subphenotypes.

Hereinafter, we discuss our results in accordance with both MCs and age-sex clusters and relate them with supporting literature to discuss the clusters’ clinical consistency through the associated risk factors, including age, habits, and comorbidities, as well as on the clusters’ sources. Finally, we present recommendations based on this study and discuss possible limitations.

#### Age

The 2 groups with very high RRs were MC2 and MC3, which included children and young adults. Age may play a protective role against the disease for 2 reasons. First, as proven by MC3 versus all single-aged groups (MCs 6-11), the incidence of pneumonia was lower in young healthy groups; hence, a good RR could be attributable to mild disease caused by SARS-CoV-2. Second, as shown by the good RRs in MC2 (children with severe disease), response to treatment is probably also better at younger ages.

In addition, children (MC2) were prioritized for medical attention compared with adults with similar clinical conditions in Mexico. After discussion with Mexican clinicians, one explanation seems to be that, at an early age, decompensation or deterioration caused by a pulmonary disease is faster in children than in adults and has a higher risk of death. In adults, there is often some time margin to evaluate the evolution of the patient’s condition before intubation or ICU admission, but the same is not true for children. Furthermore, if, in addition to the presence of pneumonia, the groups are defined by conditions such as CKD and cardiovascular issues, a child who already has those issues could be perceived as having a much higher risk or being more vulnerable than an older person. These results are supported by recent literature; for example, a study with a small cohort from Madrid [44] found 10% of 41 children with COVID-19 required ICU admission. Another study [45] showed that severe COVID-19 can also happen in small children and adolescents, in which risk factors for ICU admission included age younger than 1 month, male sex, signs of lower respiratory tract infection, and presence of a pre-existing medical condition.

Regarding the association between older age and outcomes, MCs 6-11 were exclusively composed of older adults with poor outcomes. However, overall survival cannot be explained only by age but also the presence of comorbidities and habits: Although MC11 had the highest mortality and mean age, MC7 had a similar RR with a mean age approximately 10 years younger, similar to the groups with better RRs. Besides, as widely described in the literature [46], older chronological age is not necessarily linked to higher mortality, but physiological age can be. MC1 and MC4 support this fact, since, despite containing the same number of groups of each age, they had similar RRs to the RRs of groups composed only of young adults with little incidence of previous disease (MC2, MC3) and groups composed of young adults with some frequently occurring diseases, such as diabetes and hypertension (MC5).

Of note, the clustering for the individual age-sex groups with an age >64 years revealed that centenarians (individuals of over 100 years old) repeatedly fell in the age-sex clusters with better outcomes. This fact conforms with the well-studied good health and low frailty scores [47] of this subpopulation.

#### Habits

The roles of obesity and smoking as risk factors for severe disease are complex, since they are both associated with the development of many conditions (eg, COPD [48] or cardiovascular disease [49]). In our study, the influence of obesity seems to be clear, by comparing MC4 and MC5; although both had diabetes, hypertension, and moderate RRs, they were differentiated by the fact that MC4 included patients of all ages without obesity and MC5 had mostly young adults with obesity. This seems to suggest that obese young adults may behave as “older,” implying higher mortality [46,50]. However, we found just the opposite in young individuals without pre-existing comorbidities: MC1 and MC3 had similar RRs, even though MC3 had a significant number of obese patients or smokers.

These findings suggest the role of habits cannot be considered alone but always with age, comorbidities, and duration of unhealthy habits. Our results found that smoking is a risk factor for severe COPD and cardiovascular disease, primarily in older patients (MC8, MC10, and MC11). Therefore, it is reasonable that the longer that one is a smoker, the greater the incidence of severe disease. In young patients, however, the evidence of smoking’s negative influence is not so straightforward. Some reviews have presented current smoking as a protective factor versus former smoking, while it is clearly a risk factor versus never smoking [51]. Our results showed that groups with young smokers have RRs that are not inferior to age-matched nonsmoking groups, as proven by the RR of MC3 versus that of MC2.

Regarding obesity, its influence is not so clear in older groups since all had a high ratio of certain comorbidities. Still, in young obese patients without a comorbidity (18-49M5 and 18-49F2), obesity seems unrelated to mortality.

#### Comorbidities

Diabetes and hypertension had the highest prevalences among the recorded comorbidities. Their prevalences seem to explain the decrease in RRs from MC1 and MC3 to MC4 and MC5, all of which are young adult groups. In older MCs (6-11), the results are harder to evaluate independently since both diseases were present in nearly every group, not specifically characterizing any cluster except MC9 (older patients with both diseases simultaneously alongside a low RR). These findings are in accordance with the current literature that has reported both diabetes and hypertension are independent risk factors for severe disease [46,52,53].

Immunosuppressed patients fell mostly within MC6 (older adults with diabetes, hypertension, immunosuppression, and other diseases). Of note, immunosuppressed patients were not in the clusters with the lowest RRs. This conforms with some reports that described that immunosuppression has not been confirmed as a relevant factor for disease severity, except for in patients with cancer [54,55]. MC6 also had few patients with CKD, a factor that has been studied as a key factor for disease progression [56,57], and it may be a cause for the immunosuppression in this group (odds ratio 9.65, 95% CI 9.05-10.28) according to the prevalence of immunosuppression in patients with CKD versus those without CKD.

MC7 was characterized by a high prevalence of CKD and other diseases. In this group, the RR was roughly 10% lower than in other severe subgroups. We found that CKD was highly associated with mortality and a shorter survival length. This accords with a report that revealed CKD was the factor that best explained mortality [58], implying patients with CKD could be vulnerable.

MC8 was similar to MC10 and MC11 to some extent since they all had patients with COPD. Most patients with COPD are older with comorbidities with poor outcomes, which conforms to several reviews that reported patients with COPD have an increased risk of severe pneumonia and poor outcomes when they develop COVID-19 [59,60].

Cardiovascular disease was homogeneously distributed among the groups, particularly in MC7, MC10, and MC11. Nowadays, cardiovascular disease may be a double-edged factor, since it is a proven risk factor for COVID-19 severity, but some of the treatments used, such as ACE inhibitors, have also proved to be protective against severe infections from SARS-CoV-2 [61,62].

### Assessment of Clusters’ Source Variability: State and Types of the Clinical Institution

Reliable subphenotype characterization that reflects the geographical and health care settings from which they are ascertained is crucial [63]. To date, variability in severity between Mexican states and TCIs is rarely reported [64-66] nor is variability assessed independently from age and sex. As an example, one state (eg, Morelos) may show higher severity if it includes more older and male patients, but when we compare age-sex groups, the results showed no difference in the probability of higher severity within age-sex groups of the same age range.

The interstate and TCI variability we found may be influenced by many factors such as the number and type (urban/rural) of population, sociocultural context, health care policy, quantity of medical institutions, availability of resources, and virus transmission level. Some states are more industrialized and have more economical resources (eg, Mexico City, Jalisco, the State of Mexico) than others (eg, Oaxaca, Chiapas, Guerrero). The differences found between Mexico City and the State of Mexico regarding the distribution of healthy clusters are hard to explain due to their proximity and similarities in the type of population and availability of medical resources.

One possible explanation for the differences in severity between social security institutions (IMSS and ISSSTE) and local public hospitals (SSA) is that SSA are administrated by the local states and the resources among states often differ. This phenomenon could influence these institutions’ quality and resources to attend their populations. Another supportive explanation is that, when an SSA receives severe patients and has insufficient medical resources, these patients can be transferred to the IMSS COVID-19 facilities. Consequently, this may saturate IMSS and deplete the limited resources due to an increasing number of patients, making the distribution of resources harder. These results conform with those of previous studies showing that the risk of death for an average patient attending IMSS and ISSSTE is twice the national average and 3 times higher relative to that of private clinical institutions [64]. In addition, the variability may also be explained by differences in COVID-19 testing strategies, rather than actual differences in the epidemiology of the underlying disease or population in these areas.

### Recommendations

Although a young age predisposes a patient to mild disease, we suggest that a key factor to explain the dividing line between “high,” “moderate,” and “low” RRs across all ages is using age in combination with habits and comorbidities. In addition, the relationship between the patient’s age and duration of unhealthy habits may help establish more useful prognoses and correlations.

Regarding the comorbidities that are associated with increased risk, our findings suggest that diabetes and hypertension are independent risks for severe disease and are associated with lower RRs. Patients with CKD could be more vulnerable in terms of mortality and survival length and are prone to immunosuppression. Patients with COPD are more likely to have an increased risk of severe pneumonia and poor outcomes.

The complex association between severity and patients’ sources (states or TCIs) implies a crucial socioeconomic and health care resource-level inequality. Thus, we suggest that future research should consider both state and TCI combined with MCs and age-sex subgroups (eg, using the proposed meta-clustering approach), leading to better subphenotype characterization.

As part of a surveillance system, these findings could help anticipate patients with potential poorer outcomes and help decision-making regarding vaccination priority or resource allocation. This can be important to make use of additional patient information (habits, comorbidities, sources) as well as age, in contrast to certain recommendations or policies for vaccinations based primarily on older age, profession, or social status, such as in Spain or the United States [67,68]. In fact, in some cases, such recommendations or policies might be imprecise (eg, as shown previously, higher chronological age is not necessarily linked with higher mortality; centenarians tend have a greater probability of a good outcome, and children with complex clinical pre-existing conditions may have worse outcomes than healthy older people).

### Limitations

As a possible limitation, we excluded patients who showed symptoms less than 31 days prior (ie, who were confirmed after September 30) to avoid a possible effect on the analysis of survival outcomes, which impeded us from using the most recent data that could have had changed epidemiological characteristics. In addition, the analyzed data set is public and open source, published by the Mexican government, but there is no clear statement about the source of some of the information reported by each public and private health institution and captured by the data system. The fact that more complete or accurate data might be available for those patients with more severe illness might result in differential misclassification reinforcing the clustering of factors with higher severity in some cases. In addition, requiring the patients to have laboratory-confirmed infection could result in individuals with more severe disease or acknowledged comorbidities—or other risk factors for severe outcomes—to be included in the study; however, this allowed us to focus on determining subphenotypes within this more severe population. Furthermore, the data set did not include additional relevant information about the patients who were discharged, readmitted, or vaccinated and did not include the duration of comorbidities and unhealthy habits. Further studies with population-based data regarding subphenotype characterization among discharged patients who underwent posthospitalization surveillance or were readmitted as well as the vaccinated population are highly needed.

### Conclusions

The analysis of COVID-19 subphenotypes from the proposed 2-stage cluster analysis produced a discriminative characterization and explainability over just age and sex. The resultant 11 MCs provided the bases for a deep understanding of the epidemiological and subphenotypic characterization of COVID-19 patients based on pre-existing comorbidities, habits, demographic characteristics, patient provenance, and TCIs, as well as identified the correlations between these characteristics and possible clinical outcomes of each patient-specific profile. These unbiased subphenotypes may help establish target groups for automated stratification or triage systems to support clinicians with early triage prior to further tests and laboratory results, especially in those areas where such tests are not available; prioritize vaccination among the general population; and provide the bases for planning personalized therapies or treatments.

The proposed age-sex stratification and meta-clustering technique have the potential to help design a novel data-driven model for the stratification of COVID-19 patients. In addition, the results shed light on robust conclusions about associations and causality between the subphenotypic presentation and clinical outcomes. Future studies can explore the treatment and vaccination implications, to provide guidance on clinical triage and customize therapy, and also develop clinically robust subphenotype classification methodologies combined with the proposed 2-stage cluster analysis. As the concern for efficient triage and personalized treatment increases, we facilitate further replicability of the study and generalization to data from other countries by making our experiment’s codes available.

## Appendix

Multimedia Appendix 1 Supplemental Materials.

## Abbreviations

CKD: chronic kidney disease
COPD: chronic obstructive pulmonary disease
DIF: National System for Integral Family Development
ICU: intensive care unit
IMSS: Mexican Institute of Social Security
ISSSTE: Institute for Social Security and Services for State Workers
LOESS: locally estimated scatterplot smoothing
MC: meta-cluster
MCA: multiple correspondence analysis
ML: machine learning
PCA: principal component analysis
RR: recovery rate
SEDENA: Secretariat of the National Defense
SEMAR: Secretariat of the Navy
SSA: Secretariat of Health
TIC: type of clinical institution
WHO: World Health Organization
